# A simple SNP genotyping method reveals extreme invasions of non-native haplotypes in pale chub *Opsariichthys platypus*, a common cyprinid fish in Japan

**DOI:** 10.1371/journal.pone.0191731

**Published:** 2018-01-23

**Authors:** Shigeru Kitanishi, Norio Onikura, Takahiko Mukai

**Affiliations:** 1 Faculty of Regional Studies, Gifu University, Gifu, Japan; 2 Fishery Research Laboratory, Kyushu University, Fukuoka, Japan; University of Hyogo, JAPAN

## Abstract

Biological invasion by non-native subspecies or populations is one of the most serious threats to ecosystems, because these species might be easily established in the introduced area and can negatively affect native populations through competition and hybridization. Pale chub *Opsariichthys platypus*, one of the most common fish in East Asia, exhibits clear genetic differentiation among regional populations; however, introgression and subsequent loss of genetic integrity have been occurring throughout Japan due to the artificial introduction of non-native conspecifics. In this study, we developed a simple SNP genotyping method to discriminate between native and non-native mitochondrial DNA (mtDNA) haplotypes in pale chub using real-time PCR assay. We then investigated the distribution patterns of non-native pale chub in Tokai region, located in the center of Honshu Island, Japan and developed a predictive model of the occurrence of non-natives to reveal the factors influencing their invasion. The specificity and accuracy of the genotyping method were confirmed by using samples whose haplotypes were determined previously. Extensive occurrence of non-native haplotypes in Tokai region was detected by this method. In addition, our models suggested that the presence of non-natives varied greatly depending on the river system, and was positively influenced by the impounded water areas. Our method could accurately distinguish between native and non-native haplotypes of pale chub in Japan and suggested key environmental factors associated with the presence of non-natives. This approach can greatly reduce experimental costs be a great contribution for quantitative investigation.

## Introduction

Biological invasion is one of the most serious human-induced threats to ecosystems, because invasive species can negatively affect native biodiversity and ecosystem through predation, competition, alteration of species interaction, and diffusion of diseases [1–3]. Understanding the factors affecting the successful invasion of alien species is a key issue for conservation planning and management; hence, numerous studies have been conducted focusing on invasion, such as distribution patterns, dispersal pathway, and genetic characteristics in introduced areas [3,4]. Compared to the invasive species, little attention has been paid to invasions by non-native subspecies or populations. Such non-native conspecifics might be easily established in the introduced areas and can have serious effects on the genetic integrity of native populations through hybridization, and potentially leading to reduced fitness and/or loss of locally adapted alleles [5–7].

Freshwater fish is particularly threatened by non-native conspecifics, because the chances of cryptic invasions and subsequent hybridizations are more likely to occur through intentional transplantations, inadvertent introductions, or escapees from domesticated populations [3,8]. In fact, numerous cases of invasions and hybridizations have been documented in various fish species [9–13]. In some cases, reduction in fitness of wild populations [14,15] and breakdown of evolutionary history of native populations [16] were reported. Molecular techniques, such as sequencing or fragment length analyses are powerful tools used for detecting cryptic invasions and hybridizations, because non-native conspecifics are not morphologically distinguishable from native individuals [17,18]. In addition to the detection of non-native conspecifics in populations, quantitative approach is also important because the influence of invasions might be stronger with an increasing frequency of introduced genotypes [3]. However, these methods require more time and labor because a vast number of individuals are required to analyze (for example, over thousand samples are required to analyze 30 or more individuals/locality in 40 localities) and quantitative approach have not been well implemented due to these costs. To prevent further expansion of non-native genes, loss of genetic integrity, and negative effect on native populations, development of a simple and reliable method for early detection of non-native genotypes is imperative [18].

Pale chub *Opsariichthys platypus* is a cyprinid fish distributed in East Asia, including China, Korea, and Japan, and is one of the most common fish in these areas [19]. Although pale chub exhibits clear genetic differentiation among regional populations (eastern Japan, western Japan, and Kyushu populations) [20], genetic introgression and subsequent loss of genetic integrity have been occurring throughout Japan due to the artificial introduction of pale chub, accompanied with the commercially important ayu fish *Plecoglossus altivelis* from Lake Biwa [20,21]. However, despite these threats, there are not many studies to reveal the details of the distribution of non-native conspecifics in each region or area of Japan. Given the possibility that most of the pale chub populations are mingled with artificially-introduced lineages, the genetic integrity of regional populations (i.e., evolutionary significant units of the species) will be critically endangered. Thus, regional populations that are not affected by introgression of non-native conspecifics are of primary importance for conserving the evolutionary history of the species.

Recently, there has been a growing interest in the use of single nucleotide polymorphisms (SNPs) for studies on ecology and conservation biology, including local adaptation, population structure, and individual identification (e.g., [22,23]). The development of SNP genotyping techniques has produced a great potential for developing simple inexpensive methods to discriminate between native and non-native conspecifics, despite their genetic similarity [24]. In this study, we developed a new SNP genotyping method to discriminate between native and non-native mitochondrial DNA (mtDNA) haplotypes in pale chub using real-time PCR assay, coupled with a rapid and simple DNA extraction method. We then investigated the distribution patterns of non-native pale chub in Tokai region, Japan, and developed a model predicting the invasion probability of non-native pale chubs based on river morphometrics. Although mtDNA cannot check the hybridization between native and non-native individuals, it can be used for the early screening of invaded populations in particular areas such as rivers, tributaries, and reaches.

## Materials and methods

### Ethics statement

This study was conducted with the permission of the local governments of Japan. The fish were collected under appropriate fishing licenses that allowed capture and sacrifice of the fish. The ethical approval was not required for the Institutional Animal Care and Use Committee of Gifu University, because the approval is only necessary when researchers will use reptiles, birds or mammals (according to the Gifu University Animal Experimentation Regulations). This study was carried out according to the "Guidelines for the use of fishes in research" by the Ichthyological Society of Japan (http://www.fish-isj.jp/english/guidelines.html), and the sampled animals were anesthetized so as not to suffer pain.

### Study species and sampling localities

**Fig 1:**
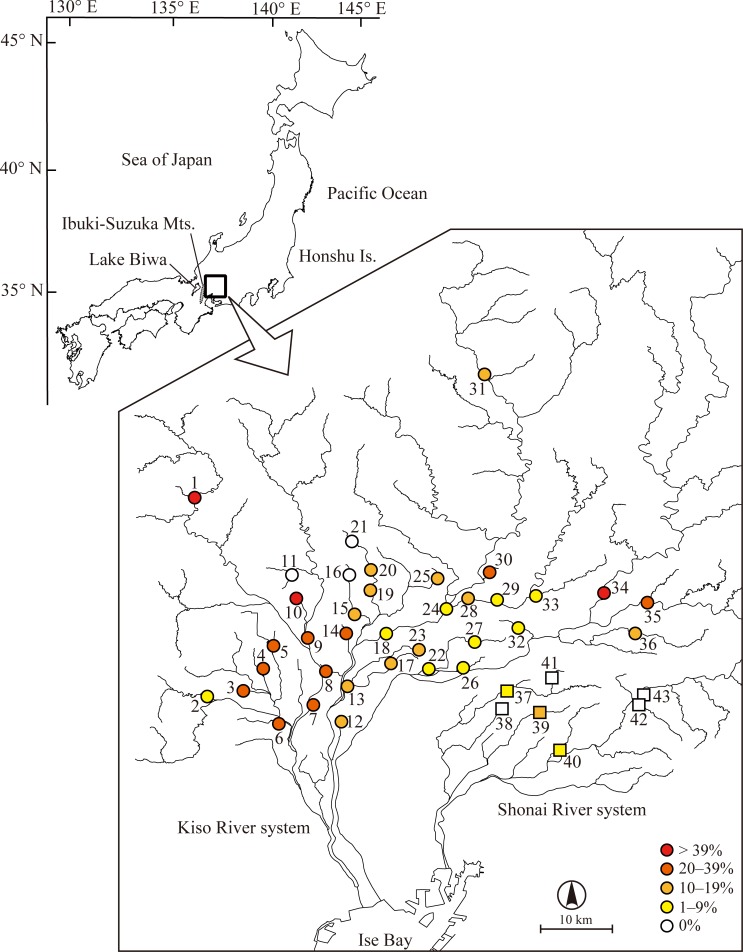
Sampling localities and presence (%) of non-native pale chub in Tokai region, Japan. Circles and squares represent localities in the Kiso River system and in the Shonai River system, respectively. For locality number, see Table 1. This figure was modified from the river data downloaded from the Geographic Information System (GIS) web page of the National Land Information Division, Japan (2008) by the authors.

Native populations of pale chub are found in the temperate rivers of western region of Japan, including Kyushu Island, Shikoku Island, and regions west of Kanto region in Honshu Island, and are one of the most common fish species in this region [19]. Recently, a phylogeographic study revealed that Japanese pale chub is divided into three major clades (eastern Japan: EJ; western Japan: WJ; and Kyushu Island: KY). The distribution of these clades, except for WJ clade, clearly reflect their respective native ranges, that is, EJ and KY are distributed in eastern Honshu (form Kanto to Tokai regions) and Kyushu Islands, respectively, and the boundary between EJ and WJ seem to correspond with the Ibuki-Suzuka Mountains (Fig 1, see also Fig 3 in [20]). However, artificial introductions of non-native populations have also been occurring mainly due to the inadvertent releases of pale chubs in conjunction with releases of ayu from Lake Biwa, located in western Japan (Fig 1) [25]. As a result, non-native haplotypes mainly comprising WJ clade have been found throughout Japan, including areas where the fish was not naturally distributed, thereby threatening native genetic types adapted to each region [20,21,25].

**Table 1:** Details of sampling localities, sample size (*N*), and results of SNP genotyping in each locality.

					SNP	
No.	Sampling localities	*N*	Lat.	Long.	EJ	WJ
Kisio River system						
1	Yokoyama Dam	31	35.626078	136.472175	0	31
2	Fujiko River	31	35.339613	136.487040	30	1
3	Doro River	30	35.346718	136.542830	24	6
4	Ohtani River	26	35.371923	136.566326	19	7
5	Oku River	31	35.403718	136.584157	20	11
6	Makita River	35	35.303770	136.594070	24	11
7	Nakasu River	31	35.315749	136.645368	24	7
8	Houe River	31	35.373856	136.663117	24	7
9	Neo River	22	35.422500	136.630253	16	6
10	Mimizu River	30	35.482806	136.613594	16	14
11	Kudase River	31	35.521657	136.613758	31	0
12	Kuwahara River	31	35.299124	136.686381	26	5
13	Nagara River (Lower)	30	35.355399	136.689332	27	3
14	Tenno River	32	35.429310	136.695093	25	7
15	Itaya River (Lower)	32	35.456191	136.708547	26	6
16	Itaya River (Upper)	31	35.514583	136.696501	31	0
17	Shin-Arata River (Lower)	29	35.381068	136.760668	25	4
18	Nagara River (Middle)	31	35.429546	136.750314	29	2
19	Ijira River (Lower)	31	35.497545	136.729812	28	3
20	Ijira River (Upper)	31	35.517308	136.733347	25	6
21	Lake Ijira	32	35.566615	136.700742	32	0
22	Shin-Sakai River (Lower)	28	35.372556	136.805804	27	1
23	Shin-Arata River (Upper)	31	35.402742	136.805906	26	5
24	Nagara River (Upper)	29	35.462151	136.837132	28	1
25	Ishida River	31	35.506689	136.825942	27	4
26	Kiso River	31	35.375802	136.858617	30	1
27	Shin-Sakai River (Upper)	32	35.417256	136.876958	31	1
28	Tsubo River	30	35.483807	136.878825	26	4
29	Seki River	32	35.494539	136.914487	30	2
30	Anonymous River	32	35.525123	136.903823	24	8
31	Nagara River (Uppermost)	30	35.810153	136.898340	25	5
32	Hazama River	32	35.438955	136.945638	31	1
33	Kawaura River	29	35.483922	136.965685	28	1
34	Kawabe Dam	30	35.496667	137.072500	17	13
35	Kaneyama Dam	32	35.475521	137.135260	24	8
36	Kani River	31	35.429791	137.117933	27	4
Shonai River system						
37	Gojo River	32	35.345105	136.930312	31	1
38	Habashita River	31	35.328433	136.930484	31	0
39	Ohyama River	31	35.311519	136.975872	26	5
40	Uchitsu River	31	35.260859	137.010145	30	1
41	Lake Iruka	31	35.368090	136.997480	31	0
42	Kasahara River	31	35.327635	137.123812	31	0
43	Toki River	32	35.331241	137.123319	32	0

Tokai region, located in the center of Honshu Island, Japan (Fig 1), is known for the high biogeographical endemicity of aquatic ecosystems and is inhabited by several endemic species (or endemic mtDNA lineages of indigenous populations), including marshy plant [26] and freshwater fish [27,28]. Although the haplotypes of clade EJ of pale chub are originally distributed in this area, non-native haplotypes (clade WJ) that possibly came from Lake Biwa are commonly found [20].

### Development of lineage-specific probes

To identify lineage-specific SNP sites that distinguish haplotypes of native (clade EJ) and non-native (clade WJ) pale chub, we obtained 162 mtDNA cytochrome *b* (cyt *b*) sequences from DNA databank DDBJ/EMBL/GenBank (Accession number: LC019793–LC019954). We then designed two probes using TaqMan minor groove binding quantification (Thermo Fisher Scientific, Waltham, MA, USA): EJ probe, 5′ FAM-CATATCAAGCCGGAATGA-NFQ 3′ (SNP site, underlined); WJ probe, 5′ VIC-CATATCAAGCCAGAATGA-NFQ 3′ (SNP site, underlined). These probes were labeled with different fluorescent dyes (FAM or VIC) as a reporter at the 5′ end and a non-fluorescent quencher dye (NFQ) at the 3′ end. A pair of primers were also designed using Primer Express software 3.0 (Thermo Fisher Scientific): forward primer, 5′-CCAGATAATTTCACTCCAGCAAACC-3′; reverse primer, 5′-CGAGTACCCCTCCTAGTTTGTTG-3′. The amplified fragment length of the PCR products was 121 bp for both haplotypes.

To verify the specificity of the designed probes and primers, a real-time TaqMan PCR for SNP genotyping was performed using 37 fish from eastern Honshu and 31 fish from western Honshu whose haplotypes were known previously and composed of major lineages within each clade (see [20]). The former samples comprised 13 haplotypes, which were collected from 7 localities of eastern Honshu including Tokai region, and the latter samples comprised 24 haplotypes that were collected from 5 localities of western Honshu, including the inlet rivers of Lake Biwa, which was thought to be the major source of non-native pale chub [19,20,25]. Genotyping was carried out using the StepOnePlus Real-Time PCR System (Thermo Fisher Scientific) in a final volume of 10 μL containing 5.0 μL of 1x TaqMan Genotyping Master Mix, 0.5 μL of 40x TaqMan SNP Genotyping Assay (8 μM of each probe and 36 μM of each primer, Thermo Fisher Scientific), and 0.5 μL of sample DNA solution. In all the genotyping assays, ultrapure water was used as the negative control. The thermal conditions were as follows: 10 min at 95°C, followed by 40 cycles at 95°C for 15 sec and at 60°C for 1 min. The data obtained were analyzed using Step One Software version 2.2.2 (Thermo Fisher Scientific).

### Sample collection, DNA extraction, and SNP genotyping

In 2015 and 2016, 1,318 pale chub individuals were collected from 43 localities of Tokai region using hand nets and casting nets (Table 1; Fig 1). Of these localities, 36 were from the Kiso River system and others were from the Shonai River system (Fig 1). All collected fish were anesthetized with 2-phenoxy ethanol, and a small piece of ventral fin was removed from each individual. Fish recovered from anesthesia were released back to the area where fish were sampled. When the juvenile fish were too small to collect their fin clip, whole fish body was preserved in 99% ethanol after euthanasia by an overdose of 2-phenoxy ethanol. Fin clips or whole body samples of juveniles were preserved in 99% ethanol until DNA extraction.

Total genomic DNA was extracted from fin using Chelex (Bio-Rad, Hercules, CA, USA) [29]. SNP genotyping for the identification of EJ and WJ haplotypes was performed using TaqMan SNP Genotyping Assay and Takara Probe PCR Mix (Takara Bio, Otsu, Japan). In the analyses using Takara Probe qPCR Mix, non-native haplotypes was labeled by HEX fluorescent dye and ROX Reference Dye was used (Takara Bio). Before SNP genotyping, repeatability of both reagents was checked against the DNA of 23 pale chub with already known haplotypes. Since the results of SNP genotyping using both reagents were the same, they were pooled for further analyses. In all the SNP genotyping assays, eight positive controls (DNA samples whose haplotypes were already identified) and a negative control (ultrapure water) were used. The real-time PCR amplifications and thermal conditions were performed according to the manufacturer’s instructions (Thermo Fisher Scientific and Takara Bio). No positive signal was obtained from any of the negative controls. The data obtained were analyzed using Step One Software.

### Model development

Correlations between the environmental factors and the occurrence of non-native haplotypes (clade WJ) were analyzed using a generalized linear model (GLM) [30]. All environmental data were downloaded from the Geographic Information System (GIS) web page of the National Land Information Division, Japan (2016). The terrain data were generated in 2009 and included the average, maximum, and minimum values for both elevation and land slope in a unit of the fourth mesh (approximately 500 × 500 m, National Land Information Division, Japan 2016). The average elevation (EL) and average land slope (SLO) were used for model analysis. The land use data of 2014, including agricultural (AA), residential (RA), forested (FA), and water surface areas (WA) were originally supplied at 1/25 size of the fourth mesh; therefore, these data were recounted in the unit of the fourth mesh. The stream data of 2009, including line and point data were collected in the unit of the fourth mesh, and the total stream length (TSL) and the number of stream connections (CON) were counted in each unit. In addition, we checked whether each mesh included water bodies impounded by dams, and transformed the data into a dichotomous variable (DAM: 1/0 with and without impounded water area) in each mesh on maps on a scale of 1:25,000. The information of the river systems of each sampling locality were also digitized into a dummy variable (SYS, 1: the Shonai River system; 0: the Kiso River system) from the information on the drainage basin provided by the same web page. The free software Quantum-GIS version 1.8.0 [31] and KASHIMIR 3D version 8.0.9 (http://www.kashmir3d.com/index-e.html) were utilized for the analysis. A correlation matrix was created for the analysis of continual variables of multicollinearity. Since EL and SLO, SLO and FA, and TSL and CON were highly correlated, as indicated by the Pearson’s correlation coefficient greater than 0.6 (Table 2), EL, FA, and TSL were excluded from the analysis to prevent multicollinearity between the predictor variables from affecting further analysis.

**Table 2:** Correlation matrix of explanatory variables used during model selection on the habitat models (Pearson’s correlation coefficient).

Variables (Acronyms)	1	2	3	4	5	6	7
1. Elevation (EL)	-						
2. Land slop (SLO)	0.630	-					
3. Total stream length (TSL)	0.230	0.254	-				
4. Number of stream connections (CON)	0.180	0.202	0.707	-			
5. Water surface area (WA)	0.103	-0.041	0.415	0.290	-		
6. Forersted area (FA)	0.547	0.816	0.178	0.225	-0.072	-	
7. Residential area (RA)	-0.058	-0.187	-0.104	-0.167	-0.146	-0.363	-
8. Agricultural area (AA)	-0.378	-0.332	-0.343	-0.260	-0.562	-0.292	-0.476

The dependent variable was the number of individuals with non-native haplotypes at each mesh, and the predictor variables included SLO, AA, RA, WA, CON, DAM, and SYS in each site on DNA analysis. In addition, square value of SLO was also used as predictor variable (SLO2) because of expressing intermediate preferences mathematically. Sample size (SIZE) was used as an offset in this analysis. A Poisson regression was conducted for all possible sets of predictor variables from a null model including no predictors to a full model including all predictors. The Akaike information criterion (AIC) [32] was used for model selection; the model with the lowest AIC was defined as the best fit model.

## Results

### Validation of lineage-specific SNP genotyping assay

The real-time PCR assay for SNP genotyping clearly identified two distinct patterns in the amplification of native (clade EJ in [20]) and non-native (clade WJ) haplotypes (S1 Fig). The amplification signals for EJ and WJ probes were observed in all 37 fish from eastern Honshu and 31 fish from western Honshu, respectively; the EJ probe did not show amplification signals in samples from western Honshu and vice versa. There was no amplification in the negative controls. Thus, we concluded that the SNP genotyping assay designed in this study could accurately discriminate between EJ and WJ haplotypes of Japanese pale chub.

### SNP genotyping of wild samples

Extensive occurrence of non-native (clade WJ) haplotypes was detected in the Tokai region using the SNP genotyping assay (Table 1; Fig 1). In the Kiso River system, many individuals had non-native haplotypes (17.8% of the individuals), and such non-native haplotypes were found in majority of the sampling localities (91.7% of the localities). In contrast to the Kiso River system, the percentage of non-native individuals in the Shonai River system was small (3.2% of the individuals) and non-native haplotypes were found only in three localities (42.9% of the localities, Table 1; Fig 1).

### Model development

The top five models are summarized in Table 3. The second and third models showed the ΔAIC of less than 2. The top three models were selected with 5 or 6 variables, which invariably included SLO, SLO2, DAM, and SYS as explanatory variables. SLO and SLO2 had positive and negative effects on the dependent variable, indicating that non-native haplotypes of pale chub were much distributed in the intermediate land slope environment in the Tokai region. DAM and SYS were positively and negatively correlated, respectively, with the dependent variable, indicating various possibilities in invasive pathway and habitat suitability of non-native populations. The predicted values calculated from the best model had a significant regression with the actual number of non-native individuals (*r* = 0.629, *P* < 0.001), indicating a fair accuracy of the model.

**Table 3:** Results of statistical analyses and selected explanatory variables of top 5 and null models for non-native population size. Acronyms of explanatory variables are according to Table 2.

				Pearson’s	Intercept	SLO	SLO2	CON	WA	RA	AA	DAM	SYS
Model	AIC	ΔAIC	wi	r					(standard error)				
1	247.50	0.00	0.179	0.629**	-1.76	0.293	-0.0598			-3.12		1.14	-1.78
					(0.15)***	(0.13)*	(0.019)**			(1.31)*		(0.18)***	(0.39)***
2	248.70	1.15	0.101	0.632**	-1.70	0.298	-0.0604		-1.16	-3.32		1.17	-1.82
					(0.16)***	(0.13)**	(0.019)**		(1.28)	(1.32)*		(0.18)***	(0.39)***
3	249.00	1.46	0.086	0.623**	-1.93	0.314	-0.0602			-2.52	0.850	1.17	-1.79
					(0.16)***	(0.13)*	(0.019)**			(1.55)	(1.17)	(0.18)***	(0.39)***
4	249.65	2.15	0.061	0.626**	-1.79	0.289	-0.0600	0.0475		-3.02		1.14	-1.79
					(0.16)***	(0.13)*	(0.019)**	(0.0933)		(1.33)*		(0.18)***	(0.39)***
5	249.76	2.26	0.058	0.591**	-2.28	0.355	-0.0614				1.93	1.22	-1.88
					(0.19)***	(0.13)**	(0.0186)***				(1.01)	(0.17)***	(0.39)***
null	332.00	82.86	0.000		1.54								
					(0.072)***								

Significant levels

***<0.001

**<0.01

*<0.05

## Discussion

We developed a unique TaqMan based SNP genotyping assay for the detection of WJ haplotypes of pale chub across Japan. The real-time PCR assay clearly identified all 68 samples whose haplotypes were previously determined by Kitanishi et al. [20] as EJ or WJ haplotypes (S1 Fig), and no misidentification was detected, suggesting that specificity of the assay is validated. Furthermore, coupled with simple DNA extraction method, the total required time from DNA extraction to PCR amplification was below 90 minutes. Previous studies have well acknowledged the specificity, robustness, and rapidity of the SNP genotyping technique (e.g. [24,33,34]) Moreover, other studies have successfully applied this technique for distinguishing closely-related organisms or genotypes in various study fields (e.g. [35–37]). Therefore, the SNP genotyping assay that is able to accurately distinguish between the EJ and WJ haplotypes of pale chub would be highly suitable for large-scale survey for monitoring the presence of non-native haplotypes in the eastern Japan.

SNP genotyping in this study revealed that non-native (clade WJ) haplotypes of pale chub were common throughout the studied area (84% of the localities). However, the dominance of non-native haplotypes varied greatly depending on the river system, and was particularly pronounced in the Kiso River system. In the Kiso River system, non-native haplotypes were observed in most of the areas (92% of the localities) and the percentage of non-natives was also high (18% of the individuals), whereas the number of localities with non-natives (43% of the localities) and the percentage of non-natives (3% of the individuals) were small in the Shonai River system. In addition, our models suggested that non-natives were likely to inhabit the Kiso River system and indicated a positive influence of the impounded water area on their presence (Table 3). These results could mainly be attributed to the inadvertent releases of non-native pale chub in conjunction with the releases of ayu from Lake Biwa. Ayu is one of the most important fishery resources in Japan, and have been released from Lake Biwa into other freshwaters throughout Japan [20]. As the Lake Biwa stock of ayu has been accompanied by other freshwater fishes, non-native populations have been introduced and expanded their distributions throughout Japan [13]. This inadvertent introduction should have occurred for pale chub. In fact, Takamura and Nakahara [21] investigated the cyt *b* sequences of 354 pale chub specimens collected from several rivers of the Kanto region, eastern Japan, and found six native (clade EJ) haplotypes from 223 specimens and 58 non-native haplotypes (clade WJ) from 131 specimens in the area. In addition, they also indicated that non-native haplotypes were considered to be the native haplotypes observed in Lake Biwa and native pale chub populations of the Kanto region have already admixed with the introduced populations. Previous studies based on a questionnaire survey on local fishermen’s cooperatives [25] and genetic studies [20,38] also reported the presence of haplotypes of WJ clade in its distribution ranges across Japan (i.e. eastern Honshu and Kyushu Islands) due to the inadvertent introduction in conjunction with ayu. Furthermore, recreational fishing of freshwater fish has been conducted in the impounded water areas; thus, artificial releases of ayu are frequently conducted by many fisheries cooperative associations in the area. In the Kiso River system, there are many impounded water areas formed by impassable dams, particularly in the main stream of the river, and there are many fisheries cooperative associations by which a vast amount (over 100 ton/year) of juvenile ayu have been released [39]. In contrast, there are few dams or weirs and only one fishery cooperative association in the Shonai River system, and fishery activities for the ayu were much lesser compared to the Kiso River system (catch volume of ayu was 0.15 ton in the Shonai River, whereas it was 460 tons in the Kiso River system, in 2015) [39].

In the present study, we found that the non-native haplotypes (clade WJ) were absent in only three and four localities of the Kiso and Shonai River systems, respectively. In the Kiso River system, the three sample localities without non-native haplotype were distributed only in the upstream of tributaries (Fig 1). The two populations (locality No. 11 and 16) were isolated from the lower rivers by several small weirs, and one population (locality No. 21) was in the agricultural reservoir, which isolates the locality from lower reaches. In the Shonai River system, the putative native populations were in the upper reaches of the main river (locality No. 42 and 43), the upstream of a tributary (locality No. 38), and agricultural reservoir (locality No. 41). These findings suggested that native populations might be preserved in small isolated areas and protected from invasion of non-native genes by the structural barriers. Although the probability of the presence of non-native haplotypes increased with the presence of dam reservoir in our models (Table 3), weirs in smaller streams or tributaries are numerous and structurally variable enough for us to prevent from using the data for the model development. These results suggested that large dams in the main rivers might induce the invasion of non-natives by releasing fish stock for fishery activities, whereas the weirs in the smaller streams might prevent the invasion from main rivers and act as a refugia for native populations [40–42].

Considering the fact that many species or populations are introduced into wild from other regions or domesticated populations [43,44], there is an increasing need for the development of rapid and reliable methods for detecting non-native conspecifics. The SNP genotyping method developed in this study could accurately discriminate between native and non-native haplotypes originated from different clades of Japanese pale chub although application of this method for haplotypes in Kyushu Island (i.e. clade KY) was not conducted. In addition, this method would assist in quantitative investigation of non-native conspecifics, similar to the discrimination of pale chub in this study. Since investigation of a large number of individuals and multiple regions to detect non-native conspecifics for conservation purpose incurs huge costs, quantitative assessment for the presence of non-native conspecifics has not been well implemented despite its wide importance [3,5]. Our genotyping method can simultaneously analyze many samples and greatly reduce the time and labor devoted to the analytical procedures, allowing quantitative evaluation of invasion essential to eradication and control programs [3]. Such investigations can screen populations that have not been influenced by non-native individuals and thus should be conserved preferentially. By using nuclear SNPs, this method could further assist in estimating the degree of hybridization between native and non-native individuals. Our approach and future development of SNP-based genotyping techniques could contribute greatly to monitor the degree of invasions and to conserve native populations.

## Supporting information

S1 Fig Example of SNP genotyping results. Discrimination plot of native (green) and non-native (blue) haplotypes of 16 pale chub samples. Solid square represents negative control (ultrapure water).
(PDF)
