# Supplementary figures and images for: A simple SNP genotyping method reveals extreme invasions of non-native haplotypes in pale chub Opsariichthys platypus, a common cyprinid fish in Japan
Source: PLoS One. 2018 Jan 23;13(1):e0191731. doi: 10.1371/journal.pone.0191731 (PMC5779690; doi:10.1371/journal.pone.0191731)

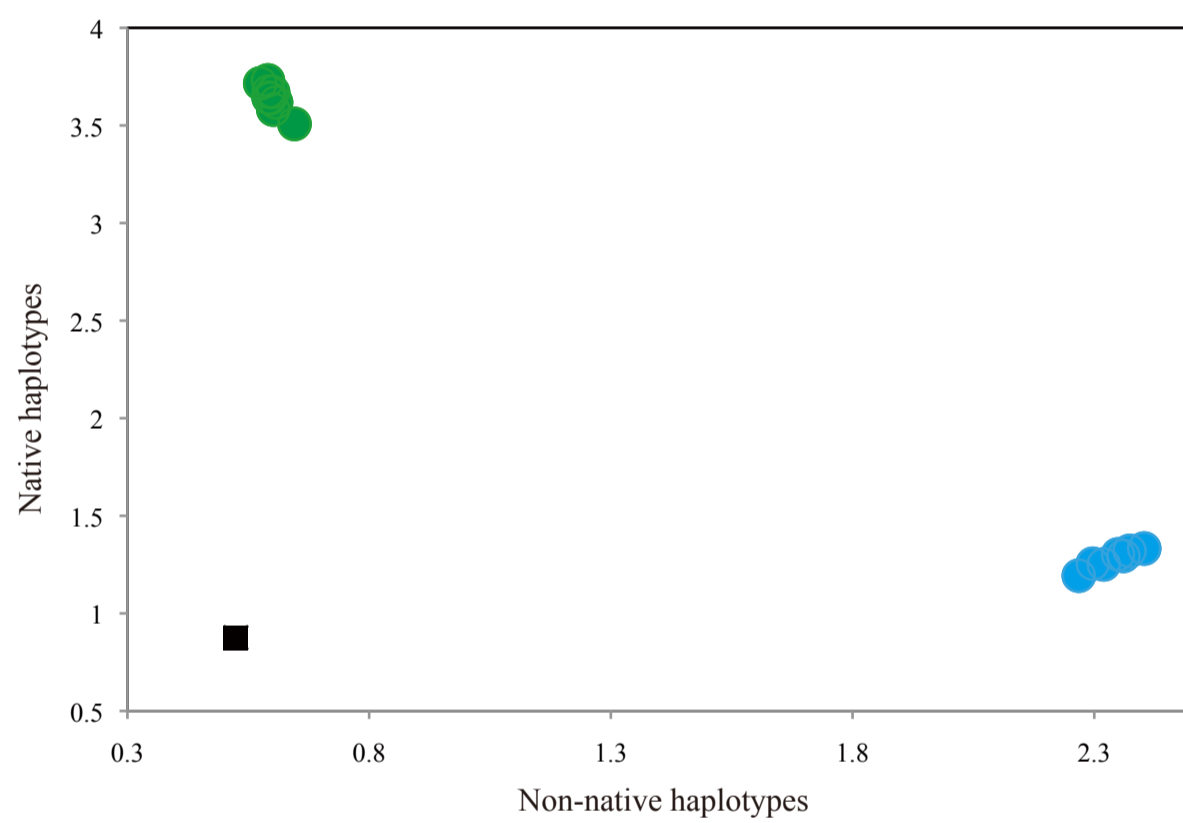

**S1 Fig**

Supplement: S1 Fig — Discrimination plot of native (green) and non-native (blue) haplotypes of 16 pale chub samples. Solid square represents negative control (ultrapure water). (PDF) [file pone.0191731.s001.pdf]
